# Supplementary material for: Population differentiation and structural variation in the Manduca sexta genome across the United States
Source: G3 (Bethesda). 2022 Feb 22;12(5):jkac047. doi: 10.1093/g3journal/jkac047 (PMC9073680; doi:10.1093/g3journal/jkac047)
Supplement: jkac047_Supplemental_Material [file jkac047_supplemental_material.docx]

Supplementary tables and figures

| Putative chromosome | Kanost *et al.* Scaffold | Gershman et al. Scaffold | Type |
| --- | --- | --- | --- |
| Z | scaffold00022 | HiC_scaffold_19 | Inversion |
| 12 | scaffold00042 | HiC_scaffold_12 | Inversion |
| 12 | scaffold00095 | HiC_scaffold_12 | Inversion |
| 12 | scaffold00119 | HiC_scaffold_12 | Inversion |
| 12 | scaffold00158 | HiC_scaffold_12 | Inversion |
| 12 | scaffold00203 | HiC_scaffold_12 | Inversion |
| 12 | scaffold00240 | HiC_scaffold_12 | Inversion |
| 12 | scaffold00264 | HiC_scaffold_12 | Inversion |
| 12 | scaffold00300 | HiC_scaffold_12 | Inversion |
| 12 | scaffold00335 | HiC_scaffold_12 | Inversion |
| 12 | scaffold00460 | HiC_scaffold_12 | Inversion |
| 12 | scaffold00466 | HiC_scaffold_12 | Inversion |
| 12 | scaffold00571 | HiC_scaffold_12 | Inversion |
| 12 | scaffold00616 | HiC_scaffold_12 | Inversion |
| 12 | scaffold00633 | HiC_scaffold_12 | Inversion |
| 12 | scaffold00790 | HiC_scaffold_12 | Inversion |
| 12 | scaffold00888 | HiC_scaffold_12 | Inversion |
| 12 | scaffold00921 | HiC_scaffold_12 | Inversion |
| 12 | scaffold00966 | HiC_scaffold_12 | Inversion |
| 12 | scaffold01138 | HiC_scaffold_12 | Inversion |
| 12 | scaffold01198 | HiC_scaffold_12 | Inversion |
| 12 | scaffold01282 | HiC_scaffold_12 | Inversion |
| 12 | scaffold01317 | HiC_scaffold_12 | Inversion |
| 12 | scaffold01366 | HiC_scaffold_12 | Inversion |
| 12 | scaffold01482 | HiC_scaffold_12 | Inversion |
| 12 | scaffold01552 | HiC_scaffold_12 | Inversion |
| 12 | scaffold00381 | HiC_scaffold_12 | Inversion,  potential pseudogene |
| 19 | scaffold00118 | HiC_scaffold_21 | Localized peak |
| 19 | Scaffold00419 | HiC_scaffold_21 | Localized peak |

**Table S1.** Scaffold information for genomic regions of interest. To be included in this table, a scaffold had to show at least one 10 kilobase window in which F_ST_ was greater than 0.5 between any two of the 3 populations. The **Type** of differentiation (e.g. localize peak vs. inversion) was determined through subsequent analyses. For convenience, genomic locations are provided for both assemblies of *M. sexta*.

| Genotype | Chr12_inversion | Stop-loss variant | Z inversion | Chr19_oddities |
| --- | --- | --- | --- | --- |
| +/+ | A36, A70, A78, A82 | A36, A70, A78, A82 | A36, A70, A71*, A78, A82, A84, A85 | A70, A71, A76, A78, A84 |
| +/- | A71, A76, A84 | A71, A76, A84 |  | A85 |
| -/- | A85 | A85 | A76* | A36, A82 |

**Table S2.** Genotypes of Arizona individuals for each of the key variants discussed in this manuscript. + denotes presence, - absence. The stop-loss variant and chromosome 12 inversion co-segregate in perfect linkage, further proof that the former resides within the latter. The Z inversion is not structured in the same way, however. Individuals marked with an * are female and hemizygous. Thus A71’s single Z carries the inversion while A76’s lacks it.


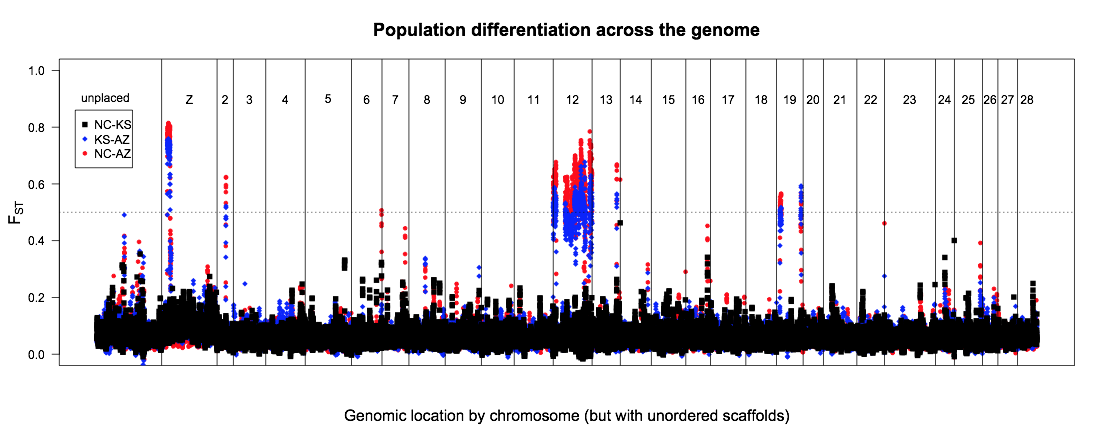


**Figure S1.** Pairwise differentiation (F_ST_) across the *M. sexta* genome using the Kanost *et al.* reference: North Carolina (NC) vs. Kansas (KS; black squares), Kansas vs. Arizona (AZ; blue diamonds), and North Carolina vs. Arizona (red points). Plotting is ordered by chromosomal linkage, but not ordered within a chromosome. We examined regions of differentiation greater than 0.5 (dashed line). By this metric, one peak on the Z, and one peak each on chromosomes 2, 13, and two on 19. Additionally, note the massive spike on chromosome 12, made up of numerous highly differentiated scaffolds. Some assignments, especially the peak on chromosome 13, were updated in the new assembly.


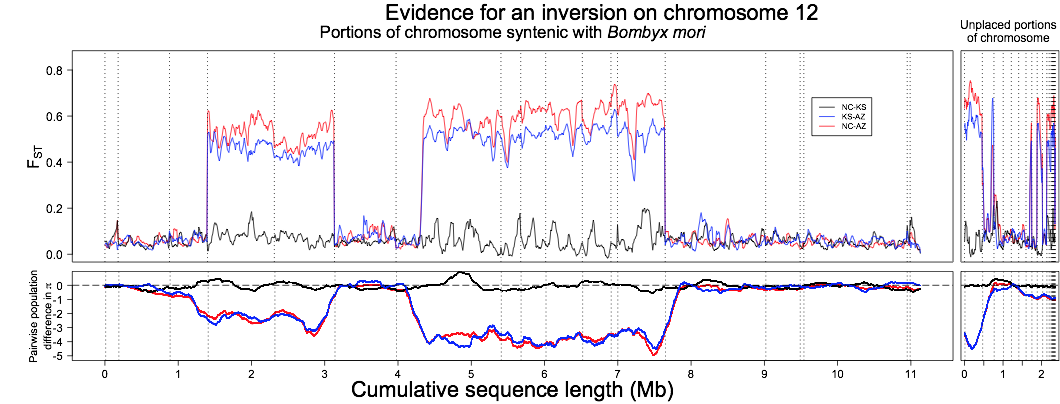


**Figure S2.** Investigation of differentiation across chromosome 12. **Top:** Ordering scaffolds from **Figure S1** via synteny with *Bombyx mori* suggests two regions > 1.5 Mb of elevated differentiation (F_ST_) between *M. sexta* populations. Given the wholly contiguous nature of the signal in the Gershman et al. assembly, this arrangement is best understood as an intra-chromosomal rearrangement between *Manduca* and *Bombyx* leading to an incorrect synteny ordering. **Bottom:** Examining deviations from background levels of variation with pairwise difference in genetic diversity (π) between populations. Diversity is roughly equal between populations (near 0 difference) except within putatively inverted regions, in which both NC and KS have fewer pairwise differences than AZ. In both the top and bottom, scaffold limits are denoted by dashed vertical lines. **Left:** The majority of assembled scaffolds could be placed in an order with synteny. **Right:** Some scaffolds could not be placed by this method and appear here, unordered. Note that some among these scaffolds also show scaffold-wide differentiation and differences in diversity. Compare to Figure 4 of the main text, in which the full length of the putative inversion appears to have been contiguously assembled.


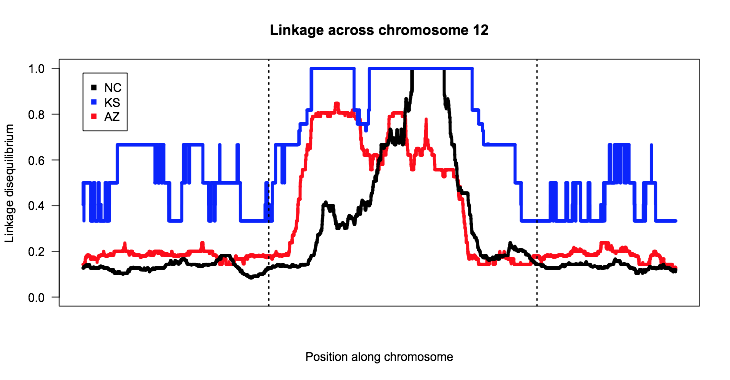


**Figure S3.** Linkage disequilibrium across chromosome 12. A rolling median of windows of 10Kb for the genotype ρ^2^ as calculated with vcftools in each population. Dashed lines indicate approximate location of the inferred inversion breakpoints. Note as well with the fewest sampled chromosomes in the Kansas population, apparent linkage is overall higher across the chromosome.


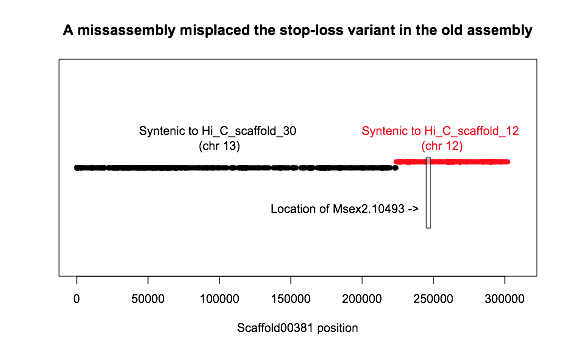


**Figure S4.** Syntenic alignment of scaffold00381 compared to the chromosome-level assembly. The majority of the scaffold (in black) is syntenic to chromosome 13, explaining its previous assignment to that chromosome. However, a significant portion of the sequence (red), including Msex2.10493, the putative segregating pseudogene, is syntenic to chromosome 12.

##
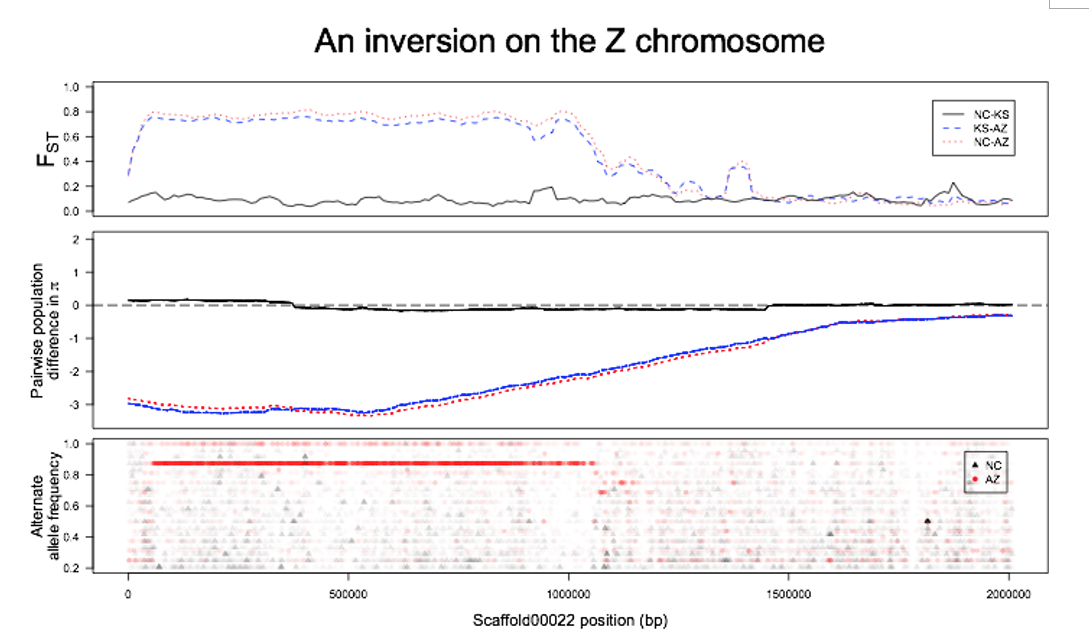


**Figure S5.** Evidence for an inversion on the Z chromosome using the Kanost et al. assembly. **Top.** A roughly 1 Mb portion of scaffold0002 (from 58 Kb to 1.03 Mb) shows elevated differentiation in North Carolina – Arizona and Kansas – Arizona comparisons. **Middle.** As with the chromosome 12 inversion, this region shows differences in π between the inverted and non-inverted region. **Bottom.** Like the chromosome 12 inversion, Arizona shows a long tract of shared allele frequencies in the region while North Carolina does not (note that Kansas allele frequencies are omitted due to lower sampling effort artificially stratifying frequencies).


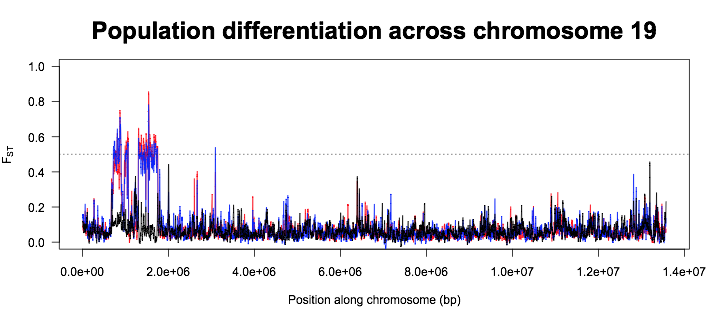


**Figure S6.** Population differentiation on chromosome 19. Two separate regions of elevated F_ST_ occur in the first two megabases of the chromosome, corresponding to the two different outlier scaffolds in the Kanost et al. assembly.
